# Addressing Weight Loss Recidivism: A Clinical Focus on Metabolic Rate and the Psychological Aspects of Obesity

**DOI:** 10.5402/2012/567530

**Published:** 2012-10-15

**Authors:** Bruce J. Grattan, Josephine Connolly-Schoonen

**Affiliations:** Department of Family Medicine, SUNY Stony Brook University Hospital Medical Center, Health Sciences Center, Level 4 Room 050, Stony Brook, NY 11794-8461, USA

## Abstract

Obesity in the United States has reached epidemic proportions and has become an unprecedented public health burden. This paper returns to the evidence for metabolic rate set points and emphasizes the clinical importance of addressing changes in metabolic rate throughout the weight loss process. In addition to the importance of clinically attending to the modulation of metabolic rate, the psychological aspects of obesity are addressed as part of the need to holistically treat obesity.

## 1. Introduction

Worldwide, approximately 310 million people are overweight or obese [1]. In the United States nearly two-thirds of the population is categorized as overweight or obese [2], with approximately 5% of the population meeting the criteria for morbid obesity [3]. Obesity accounts for an estimated 400,000 deaths per year, making its effect on mortality second only to tobacco use [4]. Current estimates even suggest a reduction in an obese individual's expected lifespan upwards of 22% [5]. Likewise, total health care costs related to obesity and comorbidities have been estimated to be 25–30% greater for obese individuals when compared with their normal weight counterparts [6, 7]. Amid such alarming statistics with extensions to both health outcomes as well as economics, in 2000, the World Health Organization proclaimed obesity to be the single greatest threat to the health of Westernized nations [8].

Given current estimates showing that 53% of Americans are currently attempting to lose weight and an additional 25% are battling weight maintenance [9], the need for appropriate dietary recommendations for both endeavors is prescient. Weight loss efforts can be considered through two lenses; initial weight loss and the maintenance of a reduced body weight. While initial weight reduction is challenging, the maintenance of such weight loss is seen as even more problematic. 

Weight lost recidivism is exemplified in a study by Sarlio-Lähteenkorva et al. who followed a cohort of 911 subjects. Only 6% maintained weight loss of at least 5% after 6 years [10]. This phenomenon has been repeatedly observed, with estimates showing that nearly one-third of weight loss is regained in the year following a weight loss intervention [11]. These finding suggest a biological drive toward weight regain and implicate the pattern of initial weight loss as a modulating factor.

## 2. Effects of Diet on Metabolic Rate

The totality of energy expenditure is comprised of three main parameters: physical activity, the thermic effect of food (TEF), and resting metabolic rate or RMR [12]. Physical activity itself is comprised of both formal and nonexercise activity thermogenesis, termed “NEAT” [13]. These parameters have been estimated to constitute 30% (physical activity), 10% (diet induced thermogenesis), and 60% (RMR) of total daily energy expenditure. RMR is largely derived from the influence of “active tissue mass”, itself related to tissue oxygen usage [14]. Basal metabolic rate (BMR) is defined as the energy requirement necessary to maintain the function of cellular processes and differs from (is lower than) the resting metabolic rate, which constitutes the energy requirement to quietly rest while awake [1]. Nevertheless, an adult of normal body weight has a BMR/kg exceeding that of an obese adult by 1 to 1.5 times [15].

Obese subjects who have been energy restricted to induce weight loss display a rapid reduction in such active tissue mass with less of a reduction in metabolically less active tissue compared with controls [16]. These values however are subject to environmental modulation. Diet composition for instance is known to directly impact the TEF, with protein consumption inducing increased caloric expenditure manifested as heat production when compared with the other macronutrients [17, 18]. Not only has the TEF been shown to be an important contributor to satiety, with protein also being the most satiating, but the TEF is known to be higher in lean subject as opposed to their obese counterparts [19]. Thus, through such means, diet composition may play an important role in weight loss. Indeed studies have confirmed this conclusion [20, 21].

As described, one of the largest contributors to daily energy expenditure is RMR. With such a large influence upon energy expenditure, diet therapy aimed at modulating these effects may prove salutary. Specific dietary alterations amenable to positive changes in RMR may be more effectual than changes in TEF.

Currently, issues regarding the concept of “RMR adaptation” [22] have not been clearly rectified, and resultantly there has not been a consensus as to the most appropriate diet therapy to minimize RMR decreases during weight loss. Garnering a thorough understanding of the factors modulating RMR is important for understanding the etiology of obesity as well as affecting sustained weight loss in overweight patients. Weight regain is not simply a volitional failure on the part of patients but may involve the recruitment of underlying survival mechanisms. 

It has been known for some time that a reduced calorie intake precipitates a reduction in metabolic rate [23, 24]. This reduction may be on the order of three times the relative reduction in weight [25, 26]. Even maintaining a 10% weight loss has been associated with reduced RMR [27]. Indeed one of the difficulties with sustained weight loss results from the reduction in metabolic rate induced by the original calorie reduction [23]. Such weight loss-induced reductions in metabolic rate and overall energy expenditure have been suggested to remain salient for up to 6 years after weight loss [28]. Likewise, a positive correlation has been observed between the degree of fat mass reduction and reductions in thermogenesis [29]. These physiological manifestations may underlie weight loss recidivism among overweight and obese subjects. Indeed, a reduction in RMR among formerly obese patients as compared with control patients who did not exhibit substantial weight loss has been found [30].

The idea of racial differences in resting metabolic rate as a driver of weight loss recidivism as well as differences in mean weight loss during intervention has been suggested in the literature [31, 32]. Such differences may be explained in behavior as opposed to biology. Although certain populations may have inherent genetic propensity towards fat storage consistent with the “thrifty gene hypothesis” [33]. These findings may also be due to differences in lean body mass distribution [34]. Issues of data interpretation are also at play. When assessed during active weight loss, a reduction in metabolic rate is expected whereas *following* weight loss such a reduction may have been “reset”. Methods to affect the extent or onset of such a reset may prove useful. 

Changes in the rate of weight loss and periods of weight maintenance/caloric adequacy may lessen the reduction in metabolic rate and promote sustained maintenance of a reduced weight. The effect of diminished RMR is also observed in those only modestly overweight (BMI 27-28) [22]. These effects are not limited to obese subjects or even overweight populations. Studies of normal weight individuals undergoing alternate day fasting show that RMR does not change under such conditions [35] indicating the importance of a return to caloric adequacy in the maintenance of metabolic rate. Quantitatively, such reductions in RMR have been seen on the order of 28% per unit of body surface area [16]. Such a significant reduction in metabolic rate, given its known influences on total daily energy expenditure makes modulation of these effects an important component to obesity treatment options. Cyclic refeeding during weight loss or the inclusion of weight maintenance breaks has been demonstrated to lessen reductions in metabolic rate. A study by Jebb et al. [36] showed that among an obese population on a very low calorie diet undergoing three cycles of diet cessation, mean absolute BMR decreased by 545, 285, and 286 kJ/day. Importantly, the only significant reduction in BMR was observed after the first study cycle. 

The reduction in RMR appears to be the result of caloric restriction rather than an intrinsic effect of reduced fat mass, as this effect was not reported following weight loss surgery once corrected for changes in fat mass and fat free mass [37, 38]. The physiological underpinnings for an evolutionary drive to reduce RMR during calorie restriction are consistent with findings of reduced activity during semistarvation in humans [39]. While convincing, not all studies have found reductions in RMR, especially when corrected for lean body mass [40, 41].

## 3. Metabolic/Body Weight Set Point

Alterations in energy intake of as little as 5% could result in changes in adiposity amounting to 6 kg annually, however body weight changes such as these are strongly resisted [42]. This example supports the idea of a biological adiposity/body weight set point, under stringent control. Despite a strong correlation between lean body mass and RMR, a 25% reduction in calorie intake has been shown to be accompanied by a reduction in resting energy expenditure which exceeds that predicted by reduction in metabolic tissue mass [43].

This set point concept has been further supported with the discovery of leptin [44], ghrelin [45], and other adipokines [46]. Despite the evidence that absolute leptin deficiency is seldom a cause of obesity, the importance of leptin in regulation of body weight remains. Elucidating the mechanisms and regulation of such a “lipostatic regulation system” [1, 47] may have a dramatic impact of the management and treatment of conditions of overweight and obesity, themselves growing at an alarming rate in the United States. Likewise, after weight-loss hyperphagia may be mediated by reductions in glucose levels as reflected in the so-called glucostatic theory [48], although this is unlikely to contribute solely. Thus, it is possible that the very benefits of weight loss with regards to improvements in lipid and carbohydrate metabolism [49] underlie weight loss recidivism to a significant degree. In explanation of these effects, it has been proposed that a reduction in insulin sensitivity promulgates a decrease in postprandial sympathetic tone resulting in a marked decrease in facultative diet induced thermogenesis among overweight and obese subjects [19]. Furthermore, it has been proposed that there exists both a memory of energy balance as well as a parallel mechanism comprised of feedback from adipose tissue [1]. Thus, the effect of maintenance breaks may help to attenuate this memory and prevent the overcompensation that would occur following transient starvation. Evidence for such memory is exemplified in a study by Wadden et al. [50]. After the first 5 weeks of energy restriction, the fall in RMR was more than double the reduction in weight, however in the same study after 48 weeks there was no difference among treatment groups. Thus, study duration may play a key role in the differences on this topic throughout the literature.

## 4. Maintenance Breaks

One potential dietary modification that may promote weight loss maintenance is the introduction of maintenance breaks. In this paradigm, subjects would initiate reduced calorie diet and after reducing their weight 1-2 BMI units, maintain this weight loss for several weeks before returning to active weight loss. Through doing so, the induction of physiological mechanisms restricting metabolic rate under conditions of reduced energy intake may be attenuated. There is little evidence for such an approach currently in the literature and such a simple alteration to the diet prescription may be of substantial benefit to subjects struggling with weight loss maintenance. Of the few citing in the literature, findings by Wing et al. [51] which suggest that maintenance breaks are not effective might be due to the duration and extent of caloric restriction in which subjects were consuming 400–500 kcal/day for 12-week periods alternating with an already restricted caloric intake of 1000–1200 kcals. Thus, a true maintenance break was not allowed as in both situations subjects were dieting, albeit varying degrees. 

A well-designed experiment of this effect was proposed by Speakman [1]. Indeed such a study has been conducted by Connolly, Trilling, Jaber, McNurlan et al. (unpublished findings) with confirmatory results as to the importance of maintenance breaks in minimizing weight loss recidivism as measured through reducing negative changes in RMR. 

## 5. Psychosocial Based Interventions

The need for individualization of obesity treatments has been recognized before [52]. Aside from the introduction of maintenance breaks to lessen changes in RMR, behavioral interventions emphasizing the psychosocial and emotional aspects of weight loss have been shown to be significantly beneficial to weight loss efforts. Such multifaceted approaches which include psychobehavioral and emotional components are supported in the literature [52].

Consistent with this holistic approach, teaching subjects to adopt effective coping strategies to actively confront stress have been shown to be an important indicator of weight loss success [53]. A study by Conradt, et al. demonstrated influences of shame and guilt on affective behavior and likewise illustrated an association between weight loss and a reduction in disengagement coping such as problem avoidance [54]. Such disengagement coping has been positively associated with anguish [55]. The psychosocial-psychosomatic interplay with eating behavior patterns and obesity is an important mediator of obesity etiology as it is understood that emotional eating is a known contributor to weight gain and obesity [56, 57]. Likewise, due to repeated failure to lose and maintain weight loss, eating among overweight individuals may coincide with a great deal of stress.

An extensive review by Ayyad and Andersen [58] demonstrated that diet in combination with group therapy is associated with a median of 27% greater long-term weight loss success. These results suggest self-efficacy and one's belief system may be instrumental to successful weight loss. Self-efficacy has long been recognized to be an important determinant in achieving health behavior change [59]. Indeed, self-efficacy has a significant impact on the degree of weight loss achievable, with one study demonstrating that self-efficacy could explain nearly 30% of the variance in weight loss effects among obese patients [60]. 

Psychosocial factors are significant contributors to weight loss recidivism [61] and may also impact the degree of initial weight loss. In a study by Dohm et al., psychosocial factors such as coping skills and self-sufficiency were stronger predictors of weight loss success than was exercise duration and intensity [61].

Numerous studies including behavioral therapies and peer support have been found to be beneficial for attenuating weight loss recidivism [62–64] and have emerged as an integral component of the weight loss prescription.

## 6. Conclusion

Amalgamating these findings, this paper suggests that the most evidence-based approach to a weight loss intervention may not only involve maintenance breaks to lessen the reduction in RMR but also, and very importantly, include holistic treatments emphasizing the psychological management of food stressors.
